# On an Epidemic of Typhoid Fever

**Published:** 1869-03

**Authors:** Robert Perry

**Affiliations:** Physician to the Glasgow Royal Infirmary


### ON AN EPIDEMIC OF TYPHOID FEVER.

By Dr. ROBERT PERRY, Physician to the Glasgow Royal Infirmary.

[During March, 1868, Dr. Perry's attention was directed to the existence of an epidemic of typhoid fever in the neighborhood of the Garnkirk Fire-Clay Company's Works, by the admission under his care into the Glasgow Royal Infirmary of five cases of that disease. The cause of the epidemic, having been investigated, was removed. The origin and mode of propagation of typhoid in isolated places such as this is, are always more instructive, and consequently more interesting, than they are in large towns.]

I may state, for the information of those who are unacquainted with the situation and surroundings of the Garnkirk Fire-Clay Works, that they are situated at about six miles from Glasgow, immediately on the line of the Caledonian Railway, from which a line of rails enters the works for the purposes connected with the manufacture of the fire-clay bricks, tiles, and many other articles, which are noted over the globe for their superior quality. Directly adjoining the works, and on ground rising somewhat towards the north and west, are a number of cottages occupied by the work-people and their families. The entire population of the surrounding houses may be roughly estimated at between 600 and 700. Beyond the workmen's cottages, and extending on every side, is an undrained moss, beneath the surface of which the famous fire-clay is found. With the exception of one or two small farm-houses and some pitmen's cottages, there are no other dwelling-houses within nearly a mile of the works. On the north side of the kilns and workshops, and surrounded almost on three sides by the cottages, is a large pond or reservoir of water for the use of the works and work-people. This reservoir is supplied with water from an adjoining clay-pit, the shaft of which is situated about 500 yards distant. The water is pumped out of the pit, and conveyed to the reservoir through fire-clay pipes, which are embedded only to a very slight depth in the ground. The number of men at work in the pit at the date of my inquiry was about fifty, and at that particular time the number was much less than the average of those who usually found employment there. I ascertained that there were no privies in the pit, and that the pitmen were in the habit of passing their evacuations, when necessary, at any convenient part of the workings. From the clayey nature of the floor of the pit, it can readily be understood that little or no absorption takes place, and the whole drainage of the pit gravitates to the bottom of the shaft, whence it is pumped up, and the water so collected passes through the before-mentioned pipes to the reservoir. The greater part of the surface drainage from the ground surrounding the cottages, as well as the sewage and waste water from the houses, is intercepted before reaching the reservoir, and carried off by open surface drains running parallel with it, and those open drains are regularly swept and kept clean. At the end of the pond, however, at which the water supply pipe is led in, I observed a considerable quantity of sewage and surface drainage finding their way into the reservoir. From this fact, and judging from the general appearance of the water in the reservoir, there could be no doubt of its being largely contaminated with sewage and putrefying matter; but, on the other hand, there is no proof that the water taken directly out of the reservoir is ever employed for drinking or cooking. At the back and side of the row of cottages, immediately adjoining where the conducting-pipe passed, I observed that the ground was thickly studded with human excrement, and refuse thrown out from the houses, the privies and dungsteads being somewhat deficient there. Workmen were, however, engaged in preparation for building them, and I understand that the deficiency has since been supplied.

In addition to other measures recommended, I advised that the joints of the conducting-pipe in the neighborhood of the cottages should all be carefully examined and made secure. I have since been informed that when this was done, one of the fire-clay pipes at this particular part was found to be broken; and, moreover, that the surface water from the ground to which I have just referred was seen to flow into the broken pipe.

Here, then, was a clear proof of the impure and unwholesome state of the water passing through the pipe; and as all the water for domestic use was drawn from this pipe as it falls into the reservoir, seeing there is no other source of supply, we need feel no surprise at the outbreak of enteric fever which took place amongst those who made daily use of it. I regret that I have not been enabled to make out the exact amount of impurities in either the pit or reservoir water, by an accurate chemical analysis. I requested a specimen of the water to be sent to me for the purpose of analyzing it; but my request was not complied with.

Throughout the cottages adjoining the works there had occurred during the past month, and up to the date of my visit, about forty-five cases of enteric fever (without being strictly accurate as to numbers). A few of the cases had been removed to the Glasgow Royal Infirmary; but the majority were under treatment in their own homes. There was also one patient suffering from enteric fever in a cottage on the opposite side of the railway, and situated only a short distance from the works; and it is an interesting fact to note, that the inmates of this cottage derived all their water-supply from the same source as the people in the works.

Mr. Murray also mentioned to me, that he had at that time one patient suffering under the same fever in the village of Chryston, which is nearly two miles from Garnkirk; but that up to the time of his seizure he was employed in the fire-clay works. I was not able to hear of any cases of the disease among the workmen of the same Company who resided at a place called Crow Row, less than half a mile distant from Garnkirk, but which is supplied with water from a different source.

It is worthy of remark, that the proprietors of the works have for years been in the habit of furnishing, gratis, a filter for the use of the inmates of each cottage. Only a few, however, of the inmates availed themselves of this offer; and it is somewhat remarkable, that I did not find a single case of fever in any of the houses where the water was regularly filtered before being used for drinking or culinary purposes. Mr. Murray subsequently informed me that one or two cases did occur among those who used the filters; but that the proportion of those attacked in the houses where filters were used was very much smaller than among those who used the water unfiltered.

Very shortly after the adoption of the measures recommended for the prevention of the contamination of the water, a marked diminution in the number of individuals attacked was observed to take place. In about a month after, the fever was almost eradicated; there being only five or six cases, and those of a much milder type, and principally confined to children. There were in all above sixty individuals attacked, and amongst these the proportion of fatal cases was very small. At the commencement of the epidemic, the type of the fever was somewhat indefinite. Bronchial symptoms were more prominent than enteric symptoms, and several of the patients were sent into the general medical wards of the Glasgow Royal Infirmary as cases of bronchitis. In a short time, however, the abdominal symptoms became more decided, and the characteristic diarrhœa, which was altogether absent in many of the earlier cases, soon assumed a very severe form.

I endeavored to trace out the history of the first case of enteric fever that had occurred at the works, and likewise if any connection could be discovered between the first and the succeeding ones. A rumor was mentioned to me of two men having recently come to the works who were at the time suffering from, or were immediately after attacked by, the fever. On strict inquiry, however, I was not able to find any foundation for this rumor.

Whether the germs of the disease were introduced from without, or originated *de novo* in some of the individuals who were using the water contaminated as I have pointed out, is not in this instance able to be proved beyond doubt. It is certain, however, that the very first case occurred in one of the cottages in the row immediately adjoining where the pipe conducting the water passed, and where I before mentioned that the privies and dungsteads were deficient.

If, as is more than probable, the intestinal discharges from this first case found their way into the pipe, the propagation of the fever by this means was most certain; and if the manager had not promptly carried out the recommendations for the suppression of this and the other likely sources of its propagation, there is but little doubt that the consequences would have been much more disastrous.—*Lancet*, *June 6*, *1868*, *p. 718*.

